# Cannabis use disorder and the future risk of cardiovascular disease in parous women: a longitudinal cohort study

**DOI:** 10.1186/s12916-020-01804-6

**Published:** 2020-11-19

**Authors:** Nathalie Auger, Gilles Paradis, Nancy Low, Aimina Ayoub, Siyi He, Brian J. Potter

**Affiliations:** 1 University of Montreal Hospital Research Centre, Montreal, Quebec Canada; 2 Institut national de santé publique du Québec, 190 Cremazie Blvd. E., Montreal, Quebec H2P 1E2 Canada; 3 Department of Epidemiology, Biostatistics, and Occupational Health, McGill University, Montreal, Quebec Canada; 4 Department of Social and Preventive Medicine, School of Public Health, University of Montreal, Montreal, Quebec Canada; 5 Department of Psychiatry, McGill University, Montreal, Quebec Canada; 6 Division of Cardiology, Department of Medicine, University of Montreal Hospital Centre, Montreal, Quebec Canada

**Keywords:** Cannabis, Cardiovascular diseases, Cerebrovascular disorders, Marijuana abuse, Myocardial infarction, Women

## Abstract

**Background:**

Cannabis use is increasing in women of reproductive age, but whether cannabis use disorders increase the long-term risk of cardiovascular disease in this population is not known. Cannabis may cause tachycardia, hypertension, cerebral vasoconstriction, and other adverse cardiovascular effects and has been associated with acute myocardial infarction and stroke. Data on the long-term effects of cannabis on the cardiovascular system are more limited. We assessed the relationship between cannabis use disorders early in life and the future risk of cardiovascular disease in women.

**Methods:**

We analyzed a longitudinal cohort of 1,247,035 pregnant women in Quebec, Canada, between 1989 and 2019. The main exposure was current or past history of cannabis use disorders at cohort entry. The main outcome measure included future hospital admission for any cardiovascular disorder during 18,998,986 person years of follow-up. We used Cox proportional hazards regression models adjusted for patient characteristics to compute hazard ratios (HR) and 95% confidence intervals (CI) for the association of cannabis use disorder with the later risk of cardiovascular hospitalization.

**Results:**

Women with cannabis use disorders had a higher incidence of cardiovascular hospitalization than unexposed women (58.4 vs. 33.6 per 10,000 person years). Cannabis use disorder was associated with 1.48 times the risk of cardiovascular hospitalization (95% CI 1.27–1.72), compared with no cannabis use disorder. The association was greater for cannabis with concomitant use of other substances (HR 1.84, 95% CI 1.53–2.21) than for cannabis alone (HR 1.30, 95% CI 0.99–1.72). Cannabis use disorder was strongly associated with hemorrhagic stroke, even with adjustment for other substance use (HR 2.08, CI 1.07–4.05).

**Conclusions:**

Cannabis use disorders may increase the long-term risk of cardiovascular disease in women, particularly hemorrhagic stroke. However, some of the excess risk may be due to concomitant use of other substances.

## Background

Cardiovascular disease is the leading cause of mortality in women [1], but prevention remains challenging as risk factors that can be targeted early in life are not fully understood. Cannabis use is increasing in women of reproductive age [2, 3], but whether women may be at risk of cardiovascular disease later in life is unknown. Cannabis affects heart rate, blood pressure, carboxyhemoglobin levels, and the cardiovascular system overall [4]. Cannabis causes reversible cerebral vasoconstriction syndrome, can trigger acute myocardial infarction and stroke, and may have long lasting effects on myocardial function, especially among regular users [4–7]. Cannabis may therefore be a risk factor for cardiovascular disease later in life.

While data suggest that cannabis may be associated with an increased risk of cardiovascular mortality among survivors of myocardial infarction [8–10], there is no evidence of an association in other populations [11]. In particular, young adults who use cannabis do not appear to be at risk of stroke or coronary heart disease later in life [11, 12]. However, cannabis doses in most studies tend to be low, and study design issues may have yielded biased results [6].

In the USA, 7% of pregnant women and 11% of nonpregnant women aged 12 to 44 years reported using cannabis between 2016 and 2017 [2]. Yet, studies of cannabis and cardiovascular disease in women of reproductive age are lacking. Most existing studies combine men and women and do not provide sex-stratified results [6]. Pregnant women who use cannabis tend to have a higher frequency of preterm birth, stillbirth, and placental abruption [13], disorders associated with future risk of cardiovascular disease [1, 14]. Thus, cannabis use during reproductive years has potential to be a marker of cardiovascular problems in the long term. Given current trends in cannabis legalization, clarifying whether women who use cannabis early in life have a greater risk of cardiovascular disease may provide opportunities for prevention. We assessed whether pregnant women with a history of cannabis use disorders had an elevated risk of cardiovascular disease over a 30-year follow-up period.

## Methods

### Study design and setting

We conducted a longitudinal cohort study of pregnant women from Quebec, Canada, using hospital data between 1989 and 2019. We included women who were admitted for deliveries or abortions. Because around 85% of women are parous by age 40, the cohort comprises the vast majority of women in the province [15]. Nearly all deliveries and a considerable proportion of abortive procedures occur in hospital in Quebec. This cohort is ideal because pregnancy represents a critical window of opportunity to tackle early cardiovascular risk factors [1, 14]. Moreover, pregnancy occurs at an age when many women already have a history of recreational cannabis use [16].

We used the Maintenance and Use of Data for the Study of Hospital Clientele registry, which includes the discharge abstracts of all hospitalized individuals in Quebec since April 1, 1989 [17, 18]. We extracted 1,247,035 women who ever delivered a live or stillborn infant or had a pregnancy with an abortive outcome (ectopic pregnancy, molar pregnancy, spontaneous, medical, or surgical abortion) between 1989 and 2016. Using medical insurance numbers, we followed the women over time beginning at their last pregnancy event through to the end of the study on March 31, 2019, to identify later hospitalizations for any cardiovascular outcome. We had up to 30 years of follow-up data after the last pregnancy.

We restricted the cohort to women with no history of cardiovascular problems at cohort entry to rule out the possibility that the association of cannabis with the future risk of cardiovascular hospitalization was due to previous cardiovascular events. We excluded women with invalid medical insurance numbers and women who died during pregnancy or delivery as we could not follow them over time.

### Cannabis use disorders

The main exposure was current or past history of cannabis use, including abuse, dependence, overdose, or poisoning any time before cohort entry. We used data from obstetric charts to identify women who used cannabis during pregnancy. Obstetric charts include cannabis use disorders that were self-reported or detected through toxicology screening [19]. We identified women with a previous history of cannabis use disorder using data from past hospitalizations prior to study entry.

We categorized cannabis use disorders based on timing (before vs. during pregnancy) and concomitant substance use (cannabis and other substance use, cannabis alone, other substance use alone, no substance use). We used diagnostic codes in the 9th and 10th revisions of the International Classification of Diseases (ICD) to capture cannabis and other substance use disorders, including alcohol, cocaine, opioids, stimulants, hallucinogens, sedatives, hypnotics, and volatile solvents (Additional file 1: Table S1) [17].

### Cardiovascular disorders

The main outcome measure was cardiovascular hospitalization. Using ICD codes [18], we captured heart disease (heart failure, myocardial infarction, other ischemic heart disease, angina, cardiac arrest, inflammatory heart disease, conduction disorder, valve disease, cardiomyopathy), pulmonary vascular disease (pulmonary embolism, other), cerebrovascular disease (ischemic stroke, hemorrhagic stroke, other), hypertension, atherosclerosis, aortic aneurysm or dissection, aneurysm of other vessels, and arterial embolism. We identified cardiovascular interventions using codes in the Canadian Classification of Diagnostic, Therapeutic, and Surgical Procedures and the Canadian Classification of Health Interventions. We included heart procedures (coronary angioplasty, coronary artery bypass graft, valve surgery, pacemaker, cardiac transplant, cardiopulmonary resuscitation, open heart resuscitation), vessel procedures (angiography, aorta surgery, intracranial surgery), and coronary care unit admission.

### Covariates

We accounted for potential confounders, including age at cohort entry (< 25, 25–34, ≥ 35 years), gravidity (1, 2, ≥ 3 pregnancies), mental illness including schizophrenia, depression, bipolar, anxiety, stress, personality disorders, and suicide attempt (yes, no), tobacco use (yes, no), comorbidity defined as preexisting or gestational diabetes, obesity, or dyslipidemia (yes, no; Additional file 1: Table S1), socioeconomic deprivation (yes, no, unspecified), place of residence (rural, urban, unspecified), and time period (1989–1997, 1998–2006, 2007–2016). Socioeconomic deprivation represented the most deprived quintile of the population, based on a composite index of income, education, and employment at the neighborhood level [18].

### Statistical analyses

We computed the incidence of cardiovascular hospitalization per 10,000 person years. Using the cumulative incidence function with death as a competing outcome, we plotted cumulative incidence curves over 30 years of follow-up for women with and without cannabis use disorders.

We used Cox proportional hazards regression models to estimate hazard ratios (HR) and 95% confidence intervals (CI) for the association of cannabis use disorder with the future risk of cardiovascular hospitalization. We adjusted regression models for age, gravidity, mental illness, tobacco use, comorbidity, socioeconomic deprivation, place of residence, and time period. Follow-up extended from the end of the last pregnancy until the first cardiovascular hospitalization, death, or the study end on March 31, 2019, depending on which event occurred first. We used the number of days since pregnancy as the time scale and the Fine and Gray method to account for the competing risk of death [20]. We censored women with no cardiovascular hospitalization by the study end.

In separate models, we assessed the timing of cannabis use and comorbid substance use disorders in relation to cardiovascular hospitalization. In models additionally adjusted for other substance use, we examined the association of cannabis use disorder with specific cardiovascular outcomes. We determined whether hazards were proportional or varied over follow-up. To do so, we estimated HRs at each year of follow-up using a linear interaction term between cannabis use and time, and plotted the trends [21].

In sensitivity analyses, we assessed the association between timing of cannabis use and specific cardiovascular outcomes. We determined whether including women with preexisting cardiovascular disease, or starting follow-up at the first pregnancy, affected the associations. We tested models with quadratic rather than linear interaction terms. We performed the analyses in SAS version 9.4 (SAS Institute Inc., Cary, NC). We received an ethics waiver from the institutional review board of the University of Montreal Hospital Centre as the data were de-identified.

## Results

The cohort comprised 1,247,035 women with 18,998,986 person years of follow-up, including 3472 women (0.3%) with a history of cannabis use disorders at cohort entry (Table 1). During follow-up, 169 women (4.9%) with cannabis use disorders were hospitalized for cardiovascular events, for an incidence of 58.4 per 10,000 person years (95% CI 50.2–67.8). In women with no cannabis use disorder, the incidence of cardiovascular hospitalization was 33.6 per 10,000 person years (95% CI 33.3–33.9).

**Table 1:** Incidence of cardiovascular hospitalizations according to baseline characteristics of women

	Total no. women	No. cardiovascular hospitalizations	Incidence per 10,000 person years (95% confidence interval)
Cannabis use disorder			
Yes	3472	169	58.4 (50.2–67.8)
No	1,243,563	63,754	33.6 (33.3–33.9)
Age, years			
< 25	160,911	6014	24.4 (23.8–25.0)
25–34	818,431	39,877	31.3 (31.0–31.6)
≥ 35	267,693	18,032	47.3 (46.7–48.0)
Gravidity			
1	510,480	30,887	36.2 (35.8–36.6)
2	465,363	20,548	30.0 (29.6–30.4)
≥ 3	271,192	12,488	34.7 (34.1–35.3)
Mental illness^a^			
Yes	39,801	2651	62.8 (60.4–65.2)
No	1,207,234	61,272	33.0 (32.7–33.2)
Other substance use disorder^b^			
Yes	13,488	1233	79.5 (75.2–84.1)
No	1,233,547	62,690	33.3 (33.0–33.5)
Tobacco use disorder			
Yes	31,424	2117	54.8 (52.5–57.2)
No	1,215,611	61,806	33.2 (32.9–33.5)
Comorbidity^c^			
Yes	119,378	9200	63.1 (61.8–64.4)
No	1,127,657	54,723	31.2 (30.9–31.5)
Socioeconomic deprivation			
Yes	238,801	13,921	39.7 (39.1–40.4)
No	954,391	45,061	31.4 (31.1–31.7)
Place of residence			
Rural	225,857	13,343	38.5 (37.8–39.1)
Urban	988,405	47,014	31.9 (31.6–32.2)
Time period			
1989–1997	419,838	43,174	41.8 (41.4–42.2)
1998–2006	322,524	14,829	28.4 (27.9–28.8)
2007–2016	504,673	5920	17.2 (16.8–17.7)
Total	1,247,035	63,923	33.6 (33.4–33.9)

^a^Schizophrenia, depression, bipolar, anxiety, stress, personality disorders, suicide attempt

^b^Alcohol, cocaine, opioids, stimulants, hallucinogens, sedatives, hypnotics, volatile solvents

^c^Diabetes, obesity, dyslipidemia

**Fig. 1:**
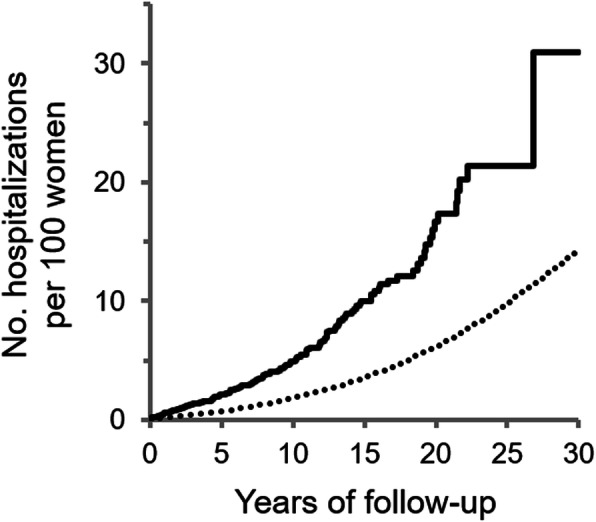
Cumulative incidence of cardiovascular hospitalization after 30 years of follow-up. Cumulative incidence of cardiovascular hospitalization per 100 women with (solid line) and without cannabis use disorder (dotted line)

After 30 years of follow-up, women with cannabis use disorders had a higher cumulative incidence of cardiovascular hospitalization (30.9 per 100 women, 95% CI 13.6–50.1) than women with no cannabis disorder (14.2 per 100 women, 95% CI 14.0–14.4) (Fig. 1). The incidence of cardiovascular hospitalization increased more sharply among women with cannabis use disorders the first few years of follow-up.

Cannabis use disorder was associated with an increased risk of cardiovascular hospitalization (Table 2). Compared with no cannabis, women with cannabis use disorders had 1.48 times the risk of future cardiovascular hospitalization (95% CI 1.27–1.72). The association was more pronounced for women with concomitant use of other substances (HR 1.84, 95% CI 1.53–2.21) than for cannabis use alone (HR 1.30, 95% CI 0.99–1.72), compared with no substance use. Use of other substances without cannabis was also associated with the risk of cardiovascular hospitalization (HR 1.81, 95% CI 1.69–1.94). Both cannabis use before and during pregnancy were associated with an increased risk of cardiovascular hospitalization.

**Table 2:** Association between cannabis use disorder and future cardiovascular hospitalization

	Total no. women	No. women with cardiovascular hospitalizations	Incidence per 10,000 person years (95% confidence interval)	Hazard ratio (95% confidence interval)	
				Unadjusted	Adjusted^a^
Cannabis use disorder					
Yes	3472	169	58.4 (50.2–67.8)	2.75 (2.36–3.20)	1.48 (1.27–1.72)
No	1,243,563	63,754	33.6 (33.3–33.9)	Referent	Referent
Comorbid substance use					
Cannabis with other substance use	1823	118	75.0 (62.7–89.9)	3.44 (2.87–4.13)	1.84 (1.53–2.21)
Cannabis alone	1649	51	38.5 (29.3–50.7)	1.97 (1.50–2.58)	1.30 (0.99–1.72)
Other substance use alone	11,665	1115	80.0 (75.5–84.9)	2.91 (2.74–3.09)	1.81 (1.69–1.94)
No substance use	1,231,898	62,639	33.3 (33.0–33.5)	Referent	Referent
Timing of cannabis use					
During pregnancy	1618	67	50.1 (39.5–63.7)	2.40 (1.88–3.05)	1.65 (1.29–2.10)
Before pregnancy	1854	102	65.4 (53.9–79.4)	3.05 (2.51–3.71)	1.38 (1.14–1.68)
No cannabis use	1,243,563	63,754	33.6 (33.3–33.9)	Referent	Referent

^a^Adjusted for age, gravidity, mental illness, tobacco use, comorbidity, socioeconomic deprivation, place of residence, and time period

In models adjusted for other substance use, cannabis use disorder was not associated with the risk of specific cardiovascular diseases, except hemorrhagic stroke (Table 3). Cannabis use disorder was associated with 2.08 times the risk of hemorrhagic stroke (95% CI 1.07–4.05), compared with no cannabis. There was no association with ischemic stroke or other cerebrovascular diseases. Women with cannabis use disorders were at greater risk of cardiovascular interventions. Cannabis use disorder was associated with 2.42 times the risk of vessel procedures (95% CI 1.21–4.87) and 1.58 times the risk of coronary care unit admission (95% CI 1.01–2.49), compared with no cannabis. There was no association with heart procedures such as coronary angioplasty.

**Table 3:** Association of cannabis use disorder with specific cardiovascular outcomes

	Cannabis use disorder		No cannabis use disorder		Hazard ratio (95% confidence interval)^a^
	No. events	Incidence per 10,000 person years	No. events	Incidence per 10,000 person years	
Cardiovascular disease	161	55.5	62,256	32.8	1.08 (0.92–1.28)
Heart					
Heart failure	10	3.4	3058	1.6	0.83 (0.43–1.58)
Myocardial infarction	9	3.0	5600	2.9	0.65 (0.33–1.26)
Other ischemic heart disease	23	7.7	10,046	5.2	1.00 (0.65–1.54)
Angina	5	1.7	2843	1.5	1.01 (0.41–2.51)
Cardiac arrest	< 5	1.3	895	0.5	0.82 (0.29–2.37)
Inflammatory heart disease	11	3.7	960	0.5	1.37 (0.72–2.61)
Conduction disorder	33	11.1	11,526	6.0	1.15 (0.80–1.64)
Valve disease	11	3.7	2692	1.4	1.34 (0.72–2.50)
Cardiomyopathy	< 5	1.3	1788	0.9	0.64 (0.23–1.81)
Lungs					
Pulmonary embolism	22	7.4	4568	2.4	1.38 (0.88–2.16)
Other pulmonary vascular disease	< 5	1.0	1236	0.6	0.67 (0.21–2.10)
Cerebrovascular					
Ischemic stroke	10	3.4	2728	1.4	1.27 (0.65–2.48)
Hemorrhagic stroke	10	3.4	2199	1.1	2.08 (1.07–4.05)
Other cerebrovascular disease	8	2.7	2241	1.2	1.22 (0.59–2.51)
Hypertension	74	25.1	39,915	20.8	0.96 (0.75–1.21)
Atherosclerosis	15	5.0	7591	3.9	0.97 (0.57–1.65)
Aortic aneurysm or dissection	< 5	0.7	360	0.2	4.61 (0.86–24.70)
Aneurysm of other vessels	< 5	0.3	1597	0.8	0.22 (0.03–1.55)
Arterial embolism	5	1.7	1019	0.5	1.37 (0.55–3.43)
Cardiovascular intervention	38	12.8	11,557	6.0	1.46 (1.04–2.05)
Heart procedure	13	4.4	6361	3.3	1.09 (0.62–1.92)
Vessel procedure	10	3.4	2979	1.5	2.42 (1.21–4.87)
Coronary care unit admission	22	7.4	4616	2.4	1.58 (1.01–2.49)

^a^Adjusted for age, gravidity, mental illness, other substance use, tobacco use, comorbidity, socioeconomic deprivation, place of residence, and time period

**Fig. 2:**
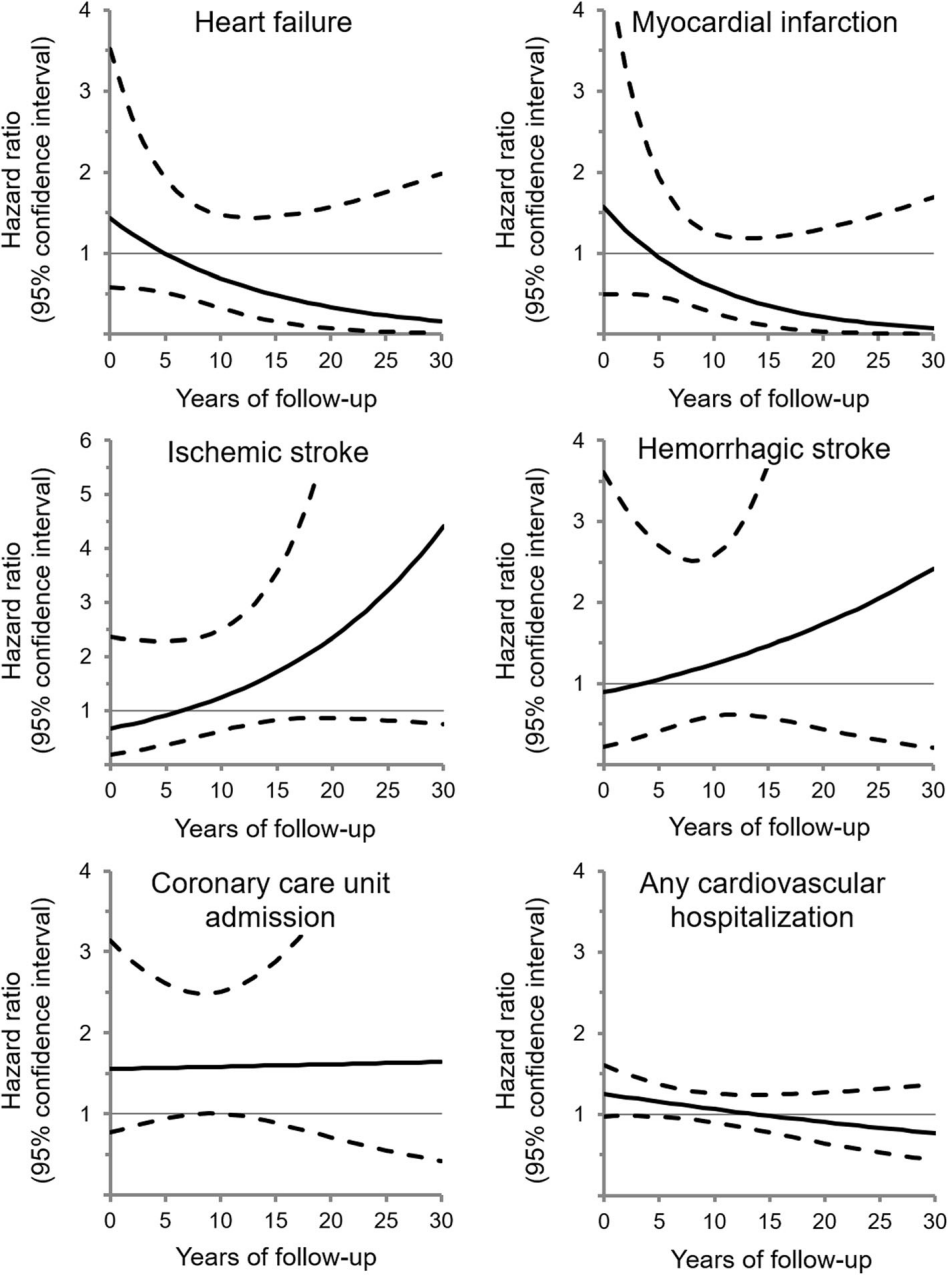
Association of cannabis use disorder with risk of cardiovascular hospitalization over time. Hazard ratio (solid line) and 95% confidence interval (dashed lines) for cannabis use disorder vs. no cannabis, adjusted for age, gravidity, mental illness, other substance use, tobacco use, comorbidity, socioeconomic deprivation, place of residence, and time period

The association of cannabis use disorders with heart failure and myocardial infarction appeared to weaken over time (Fig. 2). However, the association of cannabis use disorder with cerebrovascular diseases, including ischemic and hemorrhagic stroke, strengthened and became more apparent after 5 years of follow-up. The association with coronary care unit admission remained constant over time. In exploratory analyses, cannabis use during pregnancy was associated with a greater risk of ischemic stroke (HR 2.50, 95% CI 1.15–5.40), whereas cannabis use before pregnancy was associated with hemorrhagic stroke (HR 2.38, 95% CI 1.06–5.32), compared with no cannabis use disorder (Additional file 1: Table S2). Using quadratic rather than linear time interaction terms did not affect the trends, nor did starting follow-up at the first pregnancy or including women with preexisting cardiovascular disease.

## Discussion

In this longitudinal study with 19 million person years of follow-up, pregnant women with a history of cannabis use disorders had a weak risk of cardiovascular disease three decades later. Cannabis use was associated with 1.5 times the risk of future cardiovascular disease compared with no cannabis, although the association strengthened for concomitant use of cannabis with other substances. Cannabis use was associated with 2.1 times the risk of hemorrhagic stroke over time. Overall, the findings suggest that cannabis use disorders in women of reproductive age may increase the risk of hemorrhagic stroke and possibly other cardiovascular disease over the long term, particularly with concomitant use of other substances. Pregnant women should be made aware of the potential long-term cardiovascular effects associated with early cannabis use. Women who use cannabis or other substances may benefit from closer clinical follow-up for cardiovascular risk factors.

Little is known on the association of cannabis with the long-term risk of coronary heart disease. Although some data suggest an association with the short-term risk of myocardial infarction [4, 22], evidence from longitudinal studies is only beginning to emerge [6, 11, 23]. In a matched cohort study of 292,770 cannabis users and 10 million controls, cannabis use was associated with 1.7 times the risk of myocardial infarction during 3 years of follow-up [22]. However, the cohort had a mean age of 37.4 years, and cannabis use at younger ages could not be assessed. In a case-crossover study of 3882 patients with myocardial infarction, cannabis use in the previous year was associated with 4.8 times the risk of myocardial infarction the first hour after exposure, but the association decreased rapidly thereafter [4]. By contrast, studies with longer follow-up found no association with coronary heart disease [11, 23], similar to our results. A study of 5113 young adults with median follow-up of 27 years in the USA found no association between cannabis and risk of coronary heart disease [11]. Similarly, a study of 65,171 individuals aged 15–49 years with mean follow-up of 10 years reported no association with coronary heart disease or myocardial infarction in men or women [23]. However, cannabis exposure was likely underreported or misclassified in these studies, potentially attenuating the associations.

Several studies have reported that survivors of myocardial infarction who use cannabis are at greater risk of mortality [8–10]. In a retrospective analysis of 2097 adults with myocardial infarction and median follow-up of 11 years, cannabis was associated with two times the risk of cardiovascular and all-cause mortality [10], confirming the observations of previous studies [8, 9]. However, caution is warranted as patients with preexisting cardiovascular disease may use cannabis to treat symptoms or sequelae of myocardial infarction. These patients may already have a high baseline risk of mortality. In our analysis, associations persisted when we included women with preexisting cardiovascular disease, suggesting that use of cannabis to treat symptoms may falsely elevate the risk of cardiovascular disease.

Several cross-sectional studies suggest that cannabis use increases the acute risk of stroke, especially ischemic stroke [5, 24–26], but the long-term risk is poorly understood. A cohort study of 5113 US patients aged 18–30 years found no association with stroke during a median 27 years of follow-up [11]. Another study of 49,321 Swedish men aged 18–20 years followed over four decades reported similar findings, although a weak association with ischemic stroke seemed present [12]. These findings are however difficult to interpret as underreporting and misclassification of cannabis exposure cannot be excluded, and the risk in women was not specifically assessed. In our analysis of pregnant women, cannabis use was primarily associated with the risk of hemorrhagic stroke. The pathways leading to ischemic and hemorrhagic stroke differ. Ischemic stroke occurs when a cerebral blood vessel is obstructed, while hemorrhagic stroke is due to vessel rupture [27]. Cannabis users may be at greater risk of vessel rupture because cannabis promotes release of dopamine in the brain, increasing intracerebral blood flow [27, 28]. It is also possible that cerebral vasospasm is involved [28]. The psychoactive component of cannabis can cause vasoconstriction due to impaired cerebral blood regulation [5, 28]. However, these pathways have yet to be verified. A cross-sectional study of 937 US patients with hemorrhagic stroke found no association with cannabis use [25].

Research on other cardiovascular events is lacking. Cross-sectional data suggest that cannabis may influence the risk of heart failure [26], but we found no association in our study. A study of 3498 young adults with 25 years of follow-up in the US found that cannabis use was associated with atherosclerosis but only among tobacco smokers [29]. In our data, cannabis use disorder was not associated with atherosclerosis when we adjusted for smoking. However, adjustment may be incomplete, as we could only account for such confounders as binary variables and underreporting is possible. Smoking is a known risk factor for cardiovascular disease. Cannabis is frequently combined with tobacco, alcohol, and other illicit drugs [10, 12]. We found that women with a comorbid substance use disorder had a substantially greater risk of cardiovascular disease than women who used cannabis alone. Mental health disorders may also contribute. A recent meta-analysis of longitudinal studies reported that cannabis users under 18 years had increased risks of depression and suicidal behavior in young adulthood and suggested that risks may be greater for women than men [30]. Depression is a well-established risk factor for cardiovascular disease in women [1].

Cannabis may also have a direct impact on the cardiovascular system. Acute effects of cannabis include increased heart rate, blood pressure, vasoconstriction, and carboxyhemoglobin levels, leading to greater myocardial oxygen demand and risk of ischemia [4, 5, 8, 9]. Users of cannabis may have depressed myocardial function, including hypotension, lower cardiac contractility, and higher blood volume [7]. Cannabinoid 1 receptor is involved in insulin resistance and inflammatory responses [9, 10]. Long-term cannabis use also increases circulating levels of apolipoprotein C-III, an additional risk factor for cardiovascular disease [5].

The present study has limitations. We relied on administrative data to identify patients with self-reported cannabis use or cannabis detected through toxicology screening before cohort entry. These patients may have more severe cannabis use disorders and be more easily detected. We could not identify women with cannabis use disorders who were never admitted to hospital. Cannabis use may be underreported due to stigma. We may therefore have missed cases, particularly women with occasional cannabis use. However, mild cannabis use may be less problematic for the cardiovascular system. We could not account for changes in cannabis use after the last pregnancy, including whether women continued, quit, or initiated cannabis, and misclassification of exposure may have resulted in conservative estimates. We could not determine the type of cannabis product used and had no information on source, route, and intensity of exposure. Residual confounding may remain as data on body mass index, diet, medication use, and quantity of tobacco use were not available, and smoking may be underreported. Cannabis users are more likely to be heavy cigarette smokers. Finally, the findings may not generalize to nonpregnant women. The effect of cannabis use in nonparous women merits further research.

## Conclusions

This longitudinal study with three decades of follow-up provides novel evidence that cannabis use disorders in parous women may be associated with the future risk of cardiovascular disease, particularly when used with other substances. Cannabis use was more strongly associated with the risk of hemorrhagic stroke than other cardiovascular outcomes. More data on the long-term risk of cannabis use in women are needed to confirm these findings. Future research may benefit from testing the acute and long-term cardiovascular effects of cannabis in women of reproductive age, and the extent to which these effects mediate future onset of cardiovascular disease. In the meantime, clinicians and public health authorities should consider informing women who use cannabis of the potential risk of cardiovascular disease.

## Supplementary information

**Additional file 1: Table S1.** International Classification of Diseases (ICD) codes for exposures and covariates. **Table S2.** Association between timing of cannabis use and cardiovascular outcomes.

## Data Availability

The dataset supporting the conclusions of this article is available in the Institut de la statistique du Québec repository, [https://www.stat.gouv.qc.ca/recherche/#/accueil].

## References

[1] Garcia M, Mulvagh SL, Merz CN, Buring JE, Manson JE (2016). Cardiovascular disease in women: clinical perspectives. Circ Res.

[2] Volkow ND, Han B, Compton WM, McCance-Katz EF (2019). Self-reported medical and nonmedical cannabis use among pregnant women in the United States. JAMA..

[3] Corsi DJ, Hsu H, Weiss D, Fell DB, Walker M (2019). Trends and correlates of cannabis use in pregnancy: a population-based study in Ontario, Canada from 2012 to 2017. Can J Public Health.

[4] Mittleman MA, Lewis RA, Maclure M, Sherwood JB, Muller JE (2001). Triggering myocardial infarction by marijuana. Circulation..

[5] Rumalla K, Reddy AY, Mittal MK (2016). Recreational marijuana use and acute ischemic stroke: a population-based analysis of hospitalized patients in the United States. J Neurol Sci.

[6] Ravi D, Ghasemiesfe M, Korenstein D, Cascino T, Keyhani S (2018). Associations between marijuana use and cardiovascular risk factors and outcomes: a systematic review. Ann Intern Med.

[7] Jouanjus E, Lapeyre-Mestre M, Micallef J; French Association of the Regional Abuse and Dependence Monitoring Centres (CEIP-A) Working Group on Cannabis Complications. Cannabis use: signal of increasing risk of serious cardiovascular disorders. J Am Heart Assoc. 2014;3:e000638. 10.1161/JAHA.113.000638 PMC4187498 24760961

[8] Mukamal KJ, Maclure M, Muller JE, Mittleman MA (2008). An exploratory prospective study of marijuana use and mortality following acute myocardial infarction. Am Heart J.

[9] Frost L, Mostofsky E, Rosenbloom JI, Mukamal KJ, Mittleman MA (2013). Marijuana use and long-term mortality among survivors of acute myocardial infarction. Am Heart J.

[10] DeFilippis EM, Singh A, Divakaran S, Gupta A, Collins BL, Biery D (2018). Cocaine and marijuana use among young adults with myocardial infarction. J Am Coll Cardiol.

[11] Reis JP, Auer R, Bancks MP, Goff DC, Lewis CE, Pletcher MJ (2017). Cumulative lifetime marijuana use and incident cardiovascular disease in middle age: the Coronary Artery Risk Development in Young Adults (CARDIA) study. Am J Public Health.

[12] Falkstedt D, Wolff V, Allebeck P, Hemmingsson T, Danielsson AK (2017). Cannabis, tobacco, alcohol use, and the risk of early stroke: a population-based cohort study of 45 000 Swedish men. Stroke..

[13] Corsi DJ, Walsh L, Weiss D, Hsu H, El-Chaar D, Hawken S (2019). Association between self-reported prenatal cannabis use and maternal, perinatal, and neonatal outcomes. JAMA..

[14] Grandi SM, Filion KB, Yoon S, Ayele HT, Doyle CM, Hutcheon JA (2019). Cardiovascular disease-related morbidity and mortality in women with a history of pregnancy complications. Circulation..

[15] Martinez GM, Daniels K, Febo-Vazquez I. Fertility of men and women aged 15-44 in the United States: National Survey of Family Growth, 2011-2015. Natl Health Stat Report. 2018;113:1–17. 30248009

[16] Kann L, McManus T, Harris WA, Shanklin SL, Flint KH, Queen B (2018). Youth risk behavior surveillance — United States, 2017. MMWR Surveill Summ.

[17] Auger N, Rhéaume MA, Low N, Lee GE, Ayoub A, Luu TM. Impact of prenatal exposure to opioids, cocaine, and cannabis on eye disorders in children. J Addict Med. 2020. 10.1097/ADM.0000000000000621. 31917733

[18] Auger N, Paradis G, Healy-Profitós J, He S, Potter BJ (2020). Outcomes of takotsubo syndrome at 15 years: a matched cohort study. Am J Med..

[19] Cook JL, Green CR, de la Ronde S, Dell CA, Graves L, Morgan L (2017). Screening and management of substance use in pregnancy: a review. J Obstet Gynaecol Can.

[20] So Y, Lin G, Johnston G. Using the PHREG procedure to analyze competing-risks data. Cary, NC: SAS Institute Inc.; 2014. https://support.sas.com/rnd/app/stat/papers/2014/competingrisk2014.pdf. Accessed 30 Jan 2020.

[21] Auger N, Abrahamowicz M, Wynant W, Lo E (2014). Gestational age-dependent risk factors for preterm birth: associations with maternal education and age early in gestation. Eur J Obstet Gynecol Reprod Biol.

[22] Chami T, Kim CH (2019). Cannabis abuse and elevated risk of myocardial infarction in the young: a population-based study. Mayo Clin Proc.

[23] Sidney S (2002). Cardiovascular consequences of marijuana use. J Clin Pharmacol.

[24] Hemachandra D, McKetin R, Cherbuin N, Anstey KJ (2016). Heavy cannabis users at elevated risk of stroke: evidence from a general population survey. Aust N Z J Public Health.

[25] Westover AN, McBride S, Haley RW (2007). Stroke in young adults who abuse amphetamines or cocaine: a population-based study of hospitalized patients. Arch Gen Psychiatry.

[26] Kalla A, Krishnamoorthy PM, Gopalakrishnan A, Figueredo VM (2018). Cannabis use predicts risks of heart failure and cerebrovascular accidents: results from the National Inpatient Sample. J Cardiovasc Med (Hagerstown).

[27] Polcaro-Pichet S, Kosatsky T, Potter BJ, Bilodeau-Bertrand M, Auger N (2019). Effects of cold temperature and snowfall on stroke mortality: a case-crossover analysis. Environ Int.

[28] Rumalla K, Reddy AY, Mittal MK (2016). Association of recreational marijuana use with aneurysmal subarachnoid hemorrhage. J Stroke Cerebrovasc Dis.

[29] Auer R, Sidney S, Goff D, Vittinghoff E, Pletcher MJ, Allen NB (2018). Lifetime marijuana use and subclinical atherosclerosis: the Coronary Artery Risk Development in Young Adults (CARDIA) study. Addiction..

[30] Gobbi G, Atkin T, Zytynski T, Wang S, Askari S, Boruff J (2019). Association of cannabis use in adolescence and risk of depression, anxiety, and suicidality in young adulthood: a systematic review and meta-analysis. JAMA Psychiatry.

