# Supplementary material for: Cannabis use disorder and the future risk of cardiovascular disease in parous women: a longitudinal cohort study
Source: BMC Med. 2020 Nov 19;18:328. doi: 10.1186/s12916-020-01804-6 (PMC7677785; doi:10.1186/s12916-020-01804-6)
Supplement: Supplementary file 1 — Additional file 1: Table S1. International Classification of Diseases (ICD) codes for exposures and covariates. Table S2. Association between timing of cannabis use and cardiovascular outcomes. [file 12916_2020_1804_MOESM1_ESM.docx]

**Table S1.** International Classification of Diseases (ICD) codes for exposures and covariates

|  | ICD-9 (before 2006) | ICD-10 (beginning in 2006) |
| --- | --- | --- |
| Cannabis use | 304.3, 305.2 | F12, T40.7 |
| Other substance use |  |  |
| Alcohol | 291, 303, 305.0, 357.5, 425.5, 535.3, 571.0-571.3, 577.9, 655.4, 760.71, 790.3, 980.0, V11.3, V79.1, E860.0. E860.1 | F10, K29.2, K70, K85.2, K86.0, K86.9, O35.4, P04.3, Q86.0, T51.0, X45, X65, Y15, Z50.2, Z71.4, Z72.2, Z86.40 |
| Illicit drugs^a^ | 304.0-304.2, 304.4-304.8, 305.3-305.9, 760.70, 760.72, 760.73, 760.75, 779.5, 965.0, 967, 969.1-969.5, 969.7-969.9, 970, E850.0-E850.2, E851-E853, E854.2, E854.3, E854.8, E950.1-E950.3, E980.1-E980.3 | F11, F13-F16, F18, F19, O35.5, P96.1, R78.1, R78.2, R78.3, T40.0-T40.6, T40.8, T40.9, T42.3, T42.4, T42.6, T42.7, T43.3-T43.6, T43.8, T43.9 |
| Covariates |  |  |
| Mental illness^b^ | 293.81-293.84, 295-298, 300, 301, 306, 308, 309, 311, E950-E959 | F20-F48, F60, F61, X60-X84, Y87.0 |
| Tobacco use | 305.1, 649.0, 989.84, V15.82, E869.4 | F17, P04.2, T65.2, Z50.8, Z58.7, Z71.6, Z72.0, Z86.42 |
| Comorbidity |  |  |
| Preexisting or gestational diabetes | 249, 250, 648.0, 648.8, V12.21 | E10-E14, O24 |
| Obesity | 278.0, 649.1, V77.8 | E66 |
| Dyslipidemia | 272 | E78 |

^a^Cocaine, opioids, stimulants, hallucinogens, sedatives, hypnotics, volatile solvents

^b^Schizophrenia, depression, bipolar, anxiety, stress, personality disorders, suicide attempt

**Table S2.** Association between timing of cannabis use and cardiovascular outcomes

|  | Hazard ratio (95% confidence interval)^a^ | |
| --- | --- | --- |
|  | Cannabis use disorder during pregnancy (N=1,618) | Cannabis use disorder before pregnancy (N=1,854) |
| Cardiovascular disease | 1.31 (1.02-1.69) | 0.97 (0.79-1.19) |
| Heart |  |  |
| Heart failure | 1.42 (0.58-3.45) | 0.58 (0.24-1.43) |
| Myocardial infarction | 0.65 (0.21-2.02) | 0.65 (0.29-1.47) |
| Other ischemic heart disease | 1.23 (0.63-2.39) | 0.90 (0.52-1.55) |
| Angina | 0.65 (0.09-4.64) | 1.18 (0.43-3.30) |
| Cardiac arrest | 1.35 (0.33-5.53) | 0.59 (0.13-2.55) |
| Inflammatory heart disease | 1.46 (0.53-4.08) | 1.32 (0.60-2.92) |
| Conduction disorder | 1.21 (0.68-2.16) | 1.11 (0.72-1.73) |
| Valve disease | 1.61 (0.58-4.22) | 1.21 (0.56-2.61) |
| Cardiomyopathy | 1.39 (0.44-4.44) | 0.24 (0.03-1.76) |
| Lungs |  |  |
| Pulmonary embolism | 1.68 (0.86-3.28) | 1.22 (0.69-2.15) |
| Other pulmonary vascular disease | - | 0.98 (0.31-3.09) |
| Cerebrovascular |  |  |
| Ischemic stroke | 2.50 (1.15-5.40) | 0.58 (0.18-1.82) |
| Hemorrhagic stroke | 1.64 (0.52-5.14) | 2.38 (1.06-5.32) |
| Other cerebrovascular disease | 2.05 (0.76-5.50) | 0.86 (0.31-2.34) |
| Hypertension | 1.15 (0.79-1.67) | 0.86 (0.63-1.16) |
| Atherosclerosis | 1.26 (0.56-2.83) | 0.83 (0.42-1.65) |
| Aortic aneurysm or dissection | 5.84 (0.68-50.14) | 3.72 (0.42-33.31) |
| Aneurysm of other vessels | 0.57 (0.08-4.03) | - |
| Arterial embolism | 0.88 (0.12-6.47) | 1.60 (0.57-4.47) |
| Cardiovascular intervention | 1.55 (0.90-2.64) | 1.42 (0.93-2.16) |
| Heart procedure | 1.15 (0.47-2.81) | 1.05 (0.52-2.15) |
| Vessel procedure | 3.21 (1.29-8.03) | 1.92 (0.76-4.86) |
| Coronary care unit admission | 1.49 (0.70-3.19) | 1.63 (0.95-2.79) |

^a^Hazard ratio is for cannabis use vs. no cannabis, adjusted for age, gravidity, mental illness, other substance use, tobacco use, comorbidity, socioeconomic deprivation, place of residence, and time period
